# DPImpute: A Genotype Imputation Framework for Ultra‐Low Coverage Whole‐Genome Sequencing and its Application in Genomic Selection

**DOI:** 10.1002/advs.202412482

**Published:** 2025-02-27

**Authors:** Weigang Zheng, Wenlong Ma, Zhilong Chen, Chao Wang, Tao Sun, Wenjun Dong, Wenjing Zhang, Song Zhang, Zhonglin Tang, Kui Li, Yunxiang Zhao, Yuwen Liu

**Affiliations:** ^1^ Key Laboratory of Agricultural Animal Genetics Breeding and Reproduction of Ministry of Education & Key Lab of Swine Genetics and Breeding of Ministry of Agriculture and Rural Affairs College of Animal Science and Technology Huazhong Agricultural University Wuhan 430070 China; ^2^ Shenzhen Branch Guangdong Laboratory for Lingnan Modern Agriculture Key Laboratory of Livestock and Poultry Multi‐Omics of MARA Agricultural Genomics Institute at Shenzhen Chinese Academy of Agricultural Sciences Shenzhen 518124 China; ^3^ Innovation Group of Pig Genome Design and Breeding Research Centre for Animal Genome Agricultural Genomics Institute at Shenzhen Chinese Academy of Agricultural Sciences Shenzhen 518124 China; ^4^ State Key Laboratory of Swine and Poultry Breeding Industry National Engineering Research Center for Breeding Swine Industry Guangdong Provincial Key Lab of Agro‐Animal Genomics and Molecular Breeding College of Animal Science South China Agricultural University Guangzhou 510642 China; ^5^ Kunpeng Institute of Modern Agriculture at Foshan Chinese Academy of Agricultural Sciences Foshan 528226 China; ^6^ Guangxi Key Laboratory of Animal Breeding, Disease Control and Prevention, College of Animal Science and Technology Guangxi University Nanning 530004 China

**Keywords:** breeding models, genetic screening of embryos, genomic selection, genotype imputation, single cell genotype

## Abstract

Whole‐genome sequencing is pivotal for elucidating the complex relationships between genotype and phenotype. However, its widespread application is hindered by the high sequencing depth and large sample sizes required, especially for genomic selection (GS) reliant on precise phenotype prediction from high‐density genotype data. To address this, DPImpute (Dual‐Phase Impute) is developed, an two‐step imputation pipeline enabling accurate whole‐genome SNP genotyping under ultra‐low coverage whole‐genome sequencing (ulcWGS) depths, small testing sample sizes, and limited reference populations. DPImpute achieved 98.06% SNP imputation accuracy with minimal testing samples (≤10), reference samples (≤100), and an ultra‐low sequencing depth of 0.3X, surpassing the accuracy of existing imputation methods. Moreover, this high accuracy is maintained across multi‐ancestry human populations. Remarkably, DPImpute demonstrated accurate SNP imputation from low‐coverage sequencing data from single blood cells and single blastocyst cells, highlighting its potential in embryo GS. To enhance the accessibility of DPImpute, a user‐friendly web server (https://agdb.ecenr.com/DPImpute/home) is developed and a Docker container for seamless implementation. In summary, DPImpute can significantly expedite breeding programs through precise and cost‐effective genotyping and serve as a valuable tool for diverse population genotyping, encompassing both human and animal studies.

## Graphical abstract

DPImpute is a two‐step pipeline that outperforms existing tools in whole‐genome SNP imputation, particularly under conditions of ultra‐low coverage sequencing, small sample sizes, and limited references. It enables precise imputation for single blastocyst cells, supporting genomic selection at the pre‐implantation stage. A user‐friendly web server and Docker container are provided for seamless accessibility and integration.

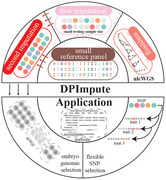

## 1 Introduction

Accurately capturing genetic variation on a genomic scale is pivotal for studying the complex associations between genotype and phenotype.^[^ ^1^, ^2^ ^]^ By precisely capturing genetic variations, researchers can pinpoint potential causal genetic variants underlying the genetic basis of complex traits, thereby unlocking opportunities to unravel the molecular mechanisms influencing these traits.^[^ ^3^, ^4^, ^5^, ^6^, ^7^, ^8^, ^9^, ^10^ ^]^ Moreover, genome‐wide genotype information enhances genetic gains in agricultural breeding by enabling genomic selection, which evaluates phenotypes based on whole‐genome SNP data.^[^ ^11^, ^12^, ^13^ ^]^ Whole‐genome sequencing (WGS) via next‐generation sequencing(NGS) provides a comprehensive and high‐resolution depiction of the entire genome, enabling the precise identification of single nucleotide polymorphisms (SNPs) as well as pivotal genetic variations such as structural variants and copy number variations. However, the expense associated with WGS and subsequent genome‐wide analysis can be prohibitive, limiting its widespread application.

Low‐coverage whole‐genome sequencing (lcWGS) combined with imputation offers a cost‐effective solution for comprehensive SNP genotyping. By leveraging linkage disequilibrium in testing samples and samples in reference panels, lcWGS can infer unsequenced genotypes, delivering extensive genomic data at reduced costs.^[^ ^14^, ^15^ ^]^ This approach enables large‐scale studies with diverse samples, supporting applications like genome‐wide association studies(GWAS) and genomic selection to uncover complex traits and enhance breeding efficiency.^[^ ^16^, ^17^, ^18^ ^]^ Additionally, genotyping preimplantation embryos for genomic selection in in vitro‐fertilized blastocysts represents a unique and significant application of imputation. This approach is especially valuable as it has the potential to enhance genetic gain at the embryonic stage while addressing the challenges of obtaining sufficient DNA from embryonic samples for comprehensive genome‐wide genotyping analyses.^[^ ^19^, ^20^ ^]^

The commonly used hidden Markov models (HMMs)‐based imputation algorithms can be categorized into three main types^[^ ^21^, ^22^, ^23^, ^24^, ^25^, ^26^, ^27^, ^28^ ^]^: First, statistical algorithms, such as BaseVar^[^ ^28^ ^]^ and STITCH,^[^ ^27^ ^]^ exploit a self‐imputation approach, which directly imputes missing genotypes from the sequencing data of the samples under testing, without the requirement of an existing reference panel. Second, Beagle,^[^ ^22^, ^23^ ^]^ QUILT,^[^ ^26^ ^]^ and IMPUTE^[^ ^24^, ^25^ ^]^ utilize the haplotype structure inherent in a high‐quality sequence reference panel to impute the missing genotypes. For example, IMPUTE introduces several key innovations, including a novel reference panel file format for rapid chromosomal access, the Positional Burrows‐Wheeler Transform (PBWT) for dynamic selection of reference haplotypes, and direct output in the BGEN file format. By utilizing PBWT, IMPUTE efficiently identifies locally matching haplotypes and long identical‐by‐state (IBS) segments as conditioning states, enabling highly accurate imputation with reduced computational demands. When paired with high‐quality reference panels, IMPUTE provides a scalable and robust solution for large‐scale genomic studies. However, it requires phased reference and testing samples without any missing alleles, which may limit its applicability in certain scenarios. Thirdly, algorithms like GLIMPSE^[^ ^21^ ^]^ combine testing samples and reference panels to infer missing genotypes. It infers haplotypes from genotype likelihoods using Gibbs sampling and PBWT to cluster haplotypes with long identical‐by‐state IBS segments, improving computational efficiency. GLIMPSE handles missing SNP data in reference panels and testing samples, making it a viable solution for low‐coverage sequencing data. However, its performance remains suboptimal for samples with sequencing depths below 1X. Besides, software such as Beagle offers flexibility by supporting genotype imputation with or without a reference panel.^[^ ^22^, ^23^ ^]^ Beagle performs both phasing and imputation tasks, emphasizing the use of long‐range haplotypic information to enhance accuracy. It is well‐suited for handling large datasets and performs particularly well when high‐quality reference panels are available, and sequencing coverage is sufficient in the testing samples. However, its accuracy diminishes in low‐coverage scenarios where a significant proportion of genotypes are missing or uncertain. In addition to imputation methods themselves, efforts such as RefRGim have focused on innovating reference panel construction methods, which have been shown to be effective in imputing low‐frequency and rare variants.^[^ ^29^ ^]^

While existing imputation frameworks address common needs, several areas still require optimization. On one hand, many widely used imputation algorithms depend heavily on a large number of reference individuals or testing samples for accurate genotype prediction.^[^ ^21^, ^24^, ^30^ ^]^ This reliance poses challenges in specific contexts, such as with newly bred strains or rare populations, where obtaining a sufficient number of samples is difficult, thus hindering precise genotype imputation. Additionally, most current imputation algorithms are designed for low‐coverage whole‐genome sequencing (lcWGS), typically at or above 0.5X coverage.^[^ ^30^, ^31^ ^]^ Given that genotyping costs remain a major concern in GWAS and breeding programs, achieving accurate imputation at even lower sequencing coverage could significantly reduce the expenses associated with whole‐genome genotyping.

In response to the aforementioned limitations, we have successfully developed a comprehensive imputation framework known as DPImpute (Dual‐Phase Impute). DPImupte overcomes the challenges of accurately inferring tens of millions of SNPs at sequencing depth lower than 0.5X, demonstrating superior imputation accuracy compared to other existing frameworks, when dealing with a limited number of testing samples (≤10) and reference samples (≤100). Additionally, we have demonstrated the effectiveness of DPImpute for single‐cell genotyping of blastocysts, further supporting its potential as a highly efficient genotyping solution for genetic screening of emobryos (GSE).

## 2 Results

### 2.1 Overview of DPImpute Framework

**Figure 1:**
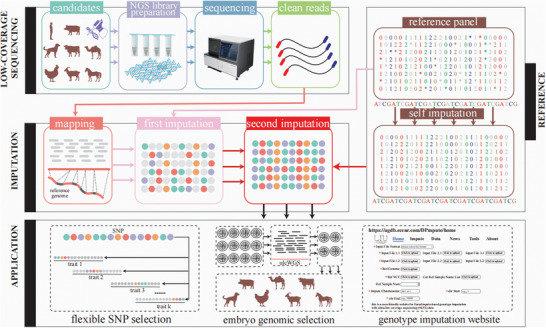
Schematic overview of DPImpute framework. The framework begins with the collection of tissue samples from the individuals under examination, followed by NGS library preparation. The subsequent sequencing can be carried out using low coverage or ultra‐low coverage sequencing. The obtained sequencing data undergoes quality control measures before being mapped to the reference genome. The resulting genotype files, in conjunction with existent high‐precision reference data, are employed as input for the initial genotype imputation round. The results from this first imputation round can then serve as the input for the second imputation round. Before the second imputation round, the reference panel undergoes self‐imputation to infer missing genotypes. This improved panel is then combined with the vcf file from the initial round for genotype refinement. These two imputation rounds, employing distinct methodologies, yield a comprehensive, high‐precision whole‐genome SNP genotype profile. The applications of the DPImpute‐based whole‐genome SNP genotype data include: the flexible selection of SNPs in genomic selection model training, the advancement of genomic selection at the embryonic stage, and animal genotype imputation website.

An overview of the DPImpute workflow is presented in **Figure** **1** DPImpute is specifically designed to accurately and efficiently impute SNPs from ultra‐low coverage whole‐genome sequencing (ulcWGS) data. Additionally, it can be applied to low coverage whole‐genome sequencing (lcWGS) data for all diploid species.

**Figure 2:**
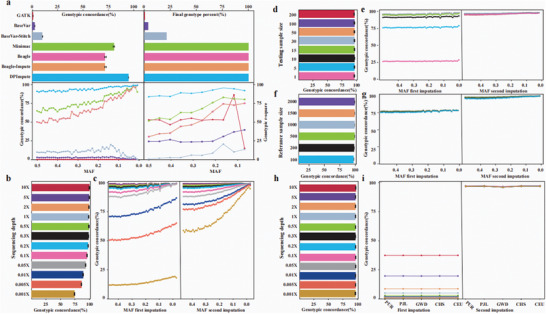
Comparison of imputation performance under different sequencing depths, testing sample sizes and reference sample sizes. a) Genotype imputation performance was evaluated across different method while maintaining a consistent sequencing depth of 0.1X for 10 testing samples. In all tests, a reference panel comprising high‐accuracy genotype data from 100 samples was utilized. Different colors represent different methods. Top left: Genotypic concordance between imputed genotypes and reference genotypes across different methods. Top right: Final genotype percentage between imputed genotypes and reference genotypes for different methods. Bottom left: Genotypic concordance of different methods after the first and second rounds of imputation, grouped by minor allele frequency (MAF). Bottom right: Genotypic r‐square of different methods, grouped by MAF. b) Genotypic concordance of genotype imputation performance was assessed across varying sequencing depths within a single pig breed population, using high‐accuracy genotype information from 2587 samples as the reference. In the 0.001X to 0.5X sequencing depth range, 200 samples were employed for testing. In the 1X to 10X sequencing depth range, the testing sample size was limited to 15 due to the scarcity of individuals with high‐coverage sequencing data. The panel shows the average genotypic concordance of DPImpute of total genotype imputation under different sequencing depth conditions. Different colors represent different sequencing depth. c) Average genotypic concordance of different sequencing depth after the first and second rounds of imputation, grouped by MAF; Different colors represent different sequencing depth same as Figure 2b. d) Genotypic concordance of genotype imputation performance was assessed across varying testing sample sizes, with a fixed sequencing depth of 0.3X for all testing samples. The reference panel size remained constant at 2587 individuals for all tests. For a thorough comparison, we conducted 20 repetitions considering inter‐sample variations when the sample size was 1. Different colors represent different testing sample sizes. e) Average genotypic concordance of different testing sample sizes after the first and second rounds of imputation, grouped by MAF; Different colors represent different testing sample sizes, consistent with Figure 2d. f) Genotypic concordance of genotype imputation performance was evaluated across different reference sample sizes, while maintaining a consistent sequencing depth of 0.3X for all testing samples. The total number of testing samples for each test was set at 5. Different colors represent different reference sample sizes. g) Average genotypic concordance of different reference sample sizes after the first and second rounds of imputation, grouped by MAF; Different colors represent different reference sample sizes same as Figure 2f. h) Genotype imputation performance was assessed across a range of sequencing depths in human multi‐ancestry populations. In all tests, high‐accuracy genotype information from a reference panel of 3000 samples was employed. The testing sample size remained constant at 30 individuals for sequencing depths spanning from 0.001X to 10X. Different colors represent different sequencing depth. i) Average genotypic concordance of different sequencing depths after the first and second rounds of imputation, grouped by MAF. Different colors represent different sequencing depth same as Figure 2h.

DPImpute implements a two‐round imputation strategy, designed to achieve accurate imputation of SNPs (Figure 1). In the initial round of imputation, genotype information for the reference population was first derived from a pre‐existing set of samples with highly accurate genotypes. GLIMPSE was then applied to the reference panel VCF and the ulcWGS data of the testing samples to construct appropriate haplotypes. These newly generated haplotypes were subsequently used to impute the SNPs of the testing samples. A key advantage of GLIMPSE is its ability to accurately impute genotypes directly from the raw sequencing data of the testing samples, which was a primary reason for selecting it for the first round.^[^ ^21^ ^]^ However, we observed that relying solely on GLIMPSE in the first round resulted in low overall genotype imputation accuracy under scenarios with small testing sample sizes and ultra‐low sequencing coverage (**Figure** **2**c,e; Figure S4, Supporting Information). This is because, in such cases, the complete absence of reads covering many SNPs prevents their genotypes from being successfully imputed in the testing samples. Nevertheless, the SNPs that were successfully imputed during this round generally demonstrated high accuracy. Although not comprehensive, these accurately imputed partial whole‐genome SNPs serve as a strong foundation for the imputation of the missing SNPs in subsequent rounds.

To address the limitation of GLIMPSE, we employed IMPUTE2 in the second round of imputation, a tool widely used for imputing genotype data from microarrays.^[^ ^24^, ^25^ ^]^ IMPUTE2 leverages the Positional Burrows‐Wheeler Transform (PBWT) on genotyped markers to identify optimal matching haplotypes and long identical‐by‐state (IBS) segments, which serve as conditioning states for the imputation model.^[^ ^25^ ^]^ It exclusively accepts input in VCF format and relies heavily on the accuracy of genotype data from the testing samples for optimal whole‐genome SNP imputation. These features of IMPUTE2 are highly compatible with the accurate genotypes for partial whole‐genome SNPs generated in the first round. It is important to note that, due to the stochastic nature of sequencing, some SNP genotypes in the reference panel may be missing.^[^ ^32^ ^]^ To address this, the reference panel was first subjected to self‐imputation using Beagle before running IMPUTE2, inferring all missing genotypes to create a thoroughly genotyped reference panel.^[^ ^22^ ^]^ This enhanced reference panel was then combined with the VCF file of the testing samples generated from the first imputation round and further refined using IMPUTE2.

In summary, the first round of imputation utilized ulcWGS sequencing data as input, accurately imputing SNPs with sufficient read coverage across all testing samples. This established a crucial foundation for the second round, which employed IMPUTE2 to genotype the missing SNPs using the VCF generated from the first round (Figure 1). Generating whole‐genome SNP genotype data with lcWGS or ulcWGS data, DPImpute enables cost‐effective genotyping, allowing for flexible SNP selection in GS models, and has the potential to advance GS to the embryonic stage.

### 2.2 Performance of DPImpute on Genotyping Compare with Other Tools for ulWGS Data

In a previous imputation framework applied to the same pig genotype dataset we utilized, the accuracy of genotype imputation using BaseVar+STITCH reached as high as 97.0% when the total sequencing depth across all samples exceeded 500X.^[^ ^33^ ^]^ However, when working with a sample size of 200 and sequencing depths of 0.1X, 0.01X, and 0.001X, BaseVar+STITCH yielded genotype imputation accuracies of only 56.66%, 10.04%, and 2.12%, respectively. In contrast, DPImpute demonstrated significantly higher accuracy, reaching 95.50%, 88.58%, and 73.89%, respectively (Figure S2a and Table S2a, Supporting Information). DPImpute did not just excel in genotype imputation accuracy under low sequencing depths but also outperformed BaseVar+STITCH when the sample size is restricted. For instance, at a sequencing depth of 0.3X, BaseVar+STITCH attained an imputation accuracy of 67.25%, 51.35%, 16.78%, 11.71%, and 4.89% for sample sizes of 100, 50, 10, 5, and 1, respectively. In contrast, DPImpute demonstrated remarkable accuracy under the same conditions, achieving 98.33%, 94.48%, 98.66%, 98.61%, and 98.29%, respectively (Figure S2b and Table S2b, Supporting Information). These results indicate that under conditions of small sample sizes and ultra‐low sequencing depths, DPImpute outperformed the original imputation software in genotype imputation accuracy.

To further benchmark the genotype imputation performance of DPImpute under conditions of a small testing sample size, a limited reference panel, and ultra‐low coverage sequencing, we compared its imputation accuracy against seven existing methods: GATK, BaseVar, BaseVar+STITCH, Minimac, Beagle, Beagle+IMPUTE, and DPImpute (Experimental Section). We randomly selected 10 samples, down‐sampled their NGS data to 0.1X for testing, and chose 100 samples to construct a reference panel (Figure 2a). Across all SNPs in the reference panel, GATK, BaseVar, BaseVar+STITCH, Minimac, Beagle, Beagle+IMPUTE, and DPImpute achieved average genotype concordance accuracies of 0.015%, 2.17%, 9.85%, 80.44%, 72.03%, 72.03%, and 94.80%, respectively (Figure 2a). For SNPs present in the reference panel, the SNP recovery rates for these methods were 0.03%, 3.66%, 21.36%, 100.00%, 100.00%, and 100.00%, respectively (Figure 2a). When focusing only on SNPs successfully genotyped by each method, the mean genotypic r‐squared values were 0.52, 0.28, 0.07, 0.68, 0.53, 0.53, and 0.90, respectively. These results highlight that DPImpute achieved the highest SNP recovery and accuracy, particularly under challenging conditions such as limited reference panel sizes, small sample sizes, and ultra‐low coverage sequencing (Figure 2a; Figure S2 and Table S2, Supporting Information).

To verify the robustness of imputation accuracy by DPImpute, we conducted five independent imputation replicates. In each replicate, 10 samples were down‐sampled to a sequencing depth of 0.1X, and 100 samples were used as reference samples. Despite the small sample size, limited reference panel, and ultra‐low sequencing depth, DPImpute achieved an average genotype imputation accuracy of 94.37%, with a standard error of 0.0078 (Figure S4, Supporting Information). Additionally, DPImpute demonstrated consistent imputation accuracy across different minor allele frequency (MAF) ranges (Figure 2a; Figure S4, Supporting Information).

### 2.3 Performance of DPImpute under Different Conditions in a Single Breed Population

We then further evaluated the imputation performance of DPImpute under varying sequencing depths, testing sample sizes, and reference sample sizes (Experimental Section).

We first assessed the genotype imputation accuracy of DPImpute across different sequencing depths for both rounds of imputation (Experimental Section, Figure 2b,c). As anticipated, imputation accuracy was exceptionally high at higher sequencing depths, with concordance rates exceeding 99.20% (99.39% at 10x and 99.29% at 5x) (Figure 2b; Figure S1 and Table S1, Supporting Information). Notably, DPImpute maintained remarkable accuracy even at lower sequencing depths (Figure 2b; Figure S1 and Table S1, Supporting Information). Notably, DPImpute maintained its superior performance even at ultra‐low coverage levels, surpassing an accuracy of 85.50% with values of 98.03%, 97.36%, 95.50%, 93.09%, 88.58%, and 85.83% for 0.3X, 0.2X, 0.1X, 0.05X, 0.01X, and 0.005X, respectively (Figure 2b; Figure S1 and Table S1, Supporting Information). Even at an extremely low coverage of 0.001X, DPImpute provided genotyping for 11.63 million SNPs with a reasonably good concordance of 73.89%.

To demonstrate the advantage of the two‐round imputation strategy implemented in DPImpute, we compared SNP imputation accuracy across a range of minor allele frequencies (MAF) before and after the second imputation round (Experimental Section, Figure 2c). Prior to the second round, accuracy was consistently high across the MAF spectrum, particularly at higher sequencing coverage levels (Figure 2c). In such scenarios, the second round of imputation provided minimal improvements in accuracy. However, its impact became increasingly significant as sequencing depth decreased, especially under ultra‐low coverage conditions. For instance, the accuracy of SNPs with a MAF of 0.5 at 0.001x coverage improved dramatically by 4.99‐fold, increasing from 11.82% to 58.99%. Similarly, for rare variants with a MAF of 0.01, accuracy experienced a remarkable 5.26‐fold improvement, rising from 18.04% to 94.97% (Figure 2c). These results underscore the effectiveness of DPImpute's two‐round imputation strategy in significantly enhancing genotype imputation accuracy, particularly under challenging ultra‐low sequencing coverage conditions.

To comprehensively evaluate the performance of DPImpute across various testing sample sizes, we conducted an assessment of imputation accuracy using a diverse range of sample sizes with a sequencing depth of 0.3X, spanning from 1 to 200 (Experimental Section, Figure 2d,e). We observed impressive imputation accuracy across different testing sample sizes with DPImpute. Specifically, the accuracy was 98.03%, 98.33%, 98.48%, 98.55%, 98.67%, 98.66%, 98.61%, and 98.29% for 200, 100, 50, 30, 15, 10, 5, and 1 sample, respectively (Figure 2d). This demonstrates that DPImpute enables accurate imputation even when working with a small number of samples, providing researchers with flexibility in data collection and enabling efficient breeding strategies.^[^ ^34^ ^]^ This advantage was majorly contributed by the second imputation round, as evidenced by the remarkable increase in accuracy from 27.80% to 98.29% when only one sample was used as input (Figure 2e).

In addition, we conducted a comparison of imputation accuracy using reference panels of different sizes (Experimental Section, Figure 2f,g). We found that, after the second imputation round, DPImpute consistently achieved high genotype imputation accuracy across reference panel sizes from 100 to 2000 (Figure 2g). When the number of reference samples decreased by 20 times, going from 2000 to 100, the imputation accuracy only exhibited a minor decrease of 1.52% (Figure 2f). Similarly, with an increase in the size of testing samples to 200, the imputation accuracy only experienced a minimal decrease of 0.86% when the reference sample size reduced from 2000 to 200, and a modest decrease of 1.05% when it decreased to 100 (Figure S3, Supporting Information).

### 2.4 Performance of DPImpute under Different Sequencing Depths in Multi‐Ancestry Human Populations

To test the robustness of DPImpute in populations with complex structures, we utilized a multi‐population dataset of the 1000 Genomes Project, which consisted of 3202 human individuals^[^ ^35^ ^]^(Experimental Section, Figure 2h,i). Genotype data from 3000 individuals were used to construct a reference panel, and SNPs were imputed for 30 randomly selected testing samples. Despite the fact that the 30 samples were drawn from five different populations and the reference panel included data from ≈26 populations, we achieved highly accurate genotype imputation results. As the sequencing coverage decreased to 1X, 0.5X, 0.1X, 0.01X, and 0.001X, the average imputation accuracy was 96.80%, 96.82%, 97.05%, 97.08%, and 96.93%, respectively (Figure 2h, Figure S5, Supporting Information). This demonstrates that DPImpute maintains accurate imputation accuracy under varying sequencing depths for multi‐ancestry human populations.

We also conducted a comparative analysis of genotype imputation performance across five human populations: PUR, PJL, GWD, CHS, and CEU (Figure 2i). Genotype imputation accuracy showed minimal variation among these populations under the same sequencing coverage and number of imputation rounds (Figure 2i). Due to the small sample size (6 samples per population), the average genotype imputation accuracy for each population was initially low after the first round (Figure 2i). However, the second round of imputation resulted in a significant improvement in accuracy across all five populations (Figure 2i; Figure S5, Supporting Information), particularly as sequencing depth decreased. For example, at a sequencing depth of 0.001X, the imputation accuracy for the PUR, PJL, GWD, CHS, and CEU populations was 96.97%, 97.15%, 96.21%, 97.18%, and 97.13%, respectively. These findings highlight the robustness and effectiveness of DPImpute in achieving high imputation accuracy, even under conditions of ultra‐low coverage and small testing sample sizes.

### 2.5 DPImpute Allows for Accurate and Consistent Phenotype Prediction Using ulWGS Data

Accurately imputing genotypes at ultra‐low sequencing depths offers significant opportunities for calculating polygenic risk scores in biomedicine and assessing genetic merit in agricultural breeding.^[^ ^33^, ^36^ ^]^ To assess the potential of DPImpute for genomic selection, we utilized both simulated and real phenotype datasets. For the simulated data, we generated 2000 traits by varying the number of causal variants (100, 500, 1000, 3000, 5000, 10000, and 20000) and combining them with four heritability levels (0.1, 0.3, 0.5, and 0.7) across 2802 pig individuals. In comparison, the real dataset consisted of seven traits derived from 1940 samples.

**Figure 3:**
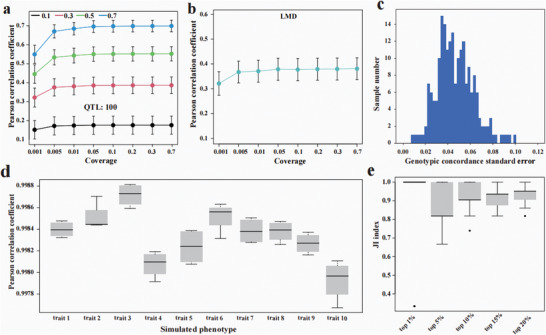
Phenotype prediction accuracy and robustness under different sequencing depths. a) PCC between genotype‐predicted phenotypes and ground ture phenotypes in the simulated data across different heritabilities with 100 QTLs. b) PCC between genotype‐predicted phenotypes and ground ture phenotypes in the real data for LMD trait. **c**) Distribution of genotypic concordance standard error across samples subjected to muletiple down‐sampling. d) The PCC of the predicted phenotypes for 200 testing samples between the first simulation round and subsequent rounds. In each round, 0.1X reads were downsampled from each testing sample and the phenotype of each sample was predicted based on a pre‐trained GS model. e) The JI distribution of the top ɑ testing samples was compared between the first simulation round and subsequent rounds. Specifically, to calculate the JI between the first and another round, we ranked the predicted phenotypic values from the first round simulation in descending order and identified the top ɑ testing samples as those with the largest phenotypic values, ranging from the 1st to the ɑth. The JI was calculated as the ratio of the number of samples identified as top ɑ by both rounds to the total number of samples identified as top ɑ by either round. For each level of top ɑ, data from 40 JIs (derived from 10 simulated traits, each with 4 JIs between the first simulation round and each subsequent simulation round) were used to create the boxplot.

Using the simulated data with 100 QTLs, we found that the prediction performance demonstrated a steady improvement as the sequencing coverage increased. Interestingly, once the sequencing coverage exceeded 0.05X, the prediction accuracy remained relatively stable (**Figure** **3**a). Indeed, our results indicate that 0.05X coverage is generally sufficient for genomic selection, outperforming the need for higher coverage such as 0.7X in most cases. Moreover, the gain in accuracy from 0.001X to 0.05X diminished with decreasing heritability. Specifically, when the heritability was as low as 0.1, sequencing at 0.005X achieved comparable prediction performance as that at 0.05X. Additionally, we observed that varying the number of QTLs had no clear effect on the prediction accuracy (Figure S6, Supporting Information), consistent with previous reports.^[^ ^37^ ^]^ Phenotype prediction using real traits demonstrated similar results. When the sequencing coverage increased from 0.001X (mean Pearson correlation coefficient (PCC) 0.319) to 0.005X (mean PCC 0.367), the relative improvement was 15.0% for loin muscle depth at 100 kg (LMD) (Figure 3b). Nevertheless, the improvement was marginal, with only a 1.1% increase observed between sequencing depths of 0.05X (0.380) and 0.7X (0.384). This trend was consistent across six other traits (Figure S7, Supporting Information).

One concern with ulWGS is the stochastic nature of SNPs covered by NGS reads for a given sample. This inherent randomness may compromise the robustness of genome‐based phenotype ranking. To assess the stability of DPImpute‐based genotyping and phenotype ranking, we simulated a scenario in which each sample underwent ulWGS multiple times. Specifically, we started with 200 samples at 0.5X coverage and randomly down‐sampled 0.1X reads five times for each sample. In each of the five rounds, we used DPImpute to genotype the samples based on the down‐sampled reads (Figure 3c). Phenotypes were predicted using rrBLUP, with a model trained with the remaining 2602 samples. The ten randomly selected traits in the training set were simulated with 0.7 heritability and 100 QTLs. Across the five rounds of simulations, the PCC of the predicted phenotypes for the 200 samples between the first round and subsequent rounds exceeded 0.99. This indicates a high consistency in sample phenotype ranking across multiple runs of ulcWGS experiments, despite the inherent stochasticity in the distribution of NGS reads under ulcWGS (Figure 3d). The Jaccard index (JI) was used to calculate the ratio of top‐ranked samples consistently recognized across simulations, with a mean JI greater than 0.84. For the top 1% of ranked samples, the JI reached as high as 0.97 (Figure 3e).

### 2.6 DPImpute Increases Phenotype Prediction Accuracy by Flexibly Including SNPs in GS Models

**Figure 4:**
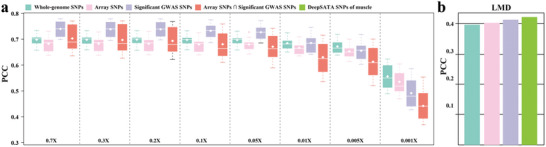
The flexibility of DPImpute in selecting SNP for GS model training increases phenotype prediction accuracy. a) Phenotype prediction accuracy of different SNP sets under different sequencing depths for simulated traits and b) at 0.7X for real LMD trait.

Unlike microarrays that typically genotype a fixed set of tens‐of‐thousands of SNP markers, DPImpute‐based genotyping encompasses a comprehensive set of SNPs present in the population, including both reference and testing samples. Thus, we hypothesized that, in addition to its low cost, another advantage of applying DPImpute to ulWGS is its potential to select a broader pool of trait‐relevant SNPs that might otherwise be unavailable on microarrays. This flexibility in SNP selection would potentially result in a higher phenotype prediction accuracy. One classic source of potentially functional SNPs arises from significant hits from GWAS analysis. Therefore, we evaluated phenotype prediction accuracy of a trait using its GWAS associated SNPs. To address this, we simulated 10 traits with 0.7 heritability and 100 QTLs from 2802 individuals. We found that, at sequencing coverage greater than or equal to 0.05X, predicting phenotypes using significant GWAS SNPs (*P*‐values < 1.0e^−12^) from whole‐genome SNPs genotyped by DPImpute achieved the best accuracy, compared to whole‐genome SNPs, SNPs on microarray, and significant GWAS SNPs on microarray (**Figure** **4**a). When considering sequencing coverages below 0.01X, we observed a decline in prediction accuracy across all four distinct SNP sets. Notably, the prediction accuracy associated with utilizing significant GWAS SNPs for phenotype prediction was surpassed by employing whole‐genome SNPs (Figure 4a).

To further demonstrate how the flexibility of DPImpute in selecting functional variants could improve genomic selection performance, we leveraged potential cis‐regulatory variants in the pig skeletal muscle to predict LMD, a phenotype highly relevant to skeletal muscle development and growth. Numerous studies have revealed that SNPs within open chromatin regions play pivotal roles in shaping complex traits by modulating enhancer activity.^[^ ^38^, ^39^ ^]^ Therefore, we analyzed pig skeletal muscle ATAC‐Seq data by DeepSATA, to quantify and rank the effects on cis‐regulatory activity of each SNP genotyped by DPImpute.^[^ ^40^ ^]^ Among these SNPs, selecting the top 32451 SNPs (the same number of array SNP) as rrBLUP input achieved the best PCC of 0.416 for predicting LMD. This performance outcompeted other SNP selections, with PCC values of 0.412 using significant GWAS SNPs (165224 SNPs with *P*‐values < 1.0e^−05^), 0.402 with whole‐genome SNPs, and 0.400 using SNPs from the microarray (Figure 4b). Taken together, our analysis indicates that ulWGS followed by DPImpute genotyping could increase the genomic selection accuracy by providing the flexibility of selecting biologically relevant SNPs in phenotype prediction.

### 2.7 DPImpute Might Assist GS at Embryonic Stage Using Single‐Cell Low‐Coverage Sequencing Data

**Figure 5:**
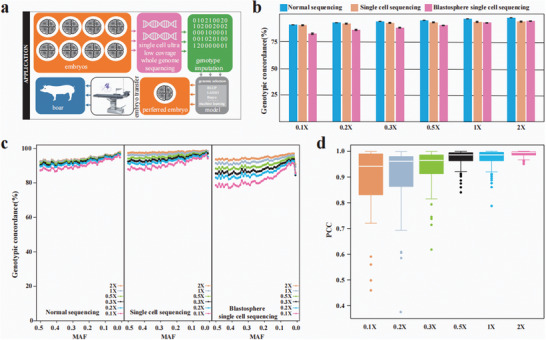
Imputation performance of single cell WGS data. a) An illustrative diagram showing how DPImpute‐based genotyping could facilitate genetic improvement of animals through genomic screening of embryos followed by phenotype prediction. b) Imputation performance under different sequencing depths for single cell sequencing and normal bulk WGS. The reference panel comprised high‐accuracy genotype information from 2587 Duroc individuals. c) Imputation performance under different sequencing depths by MAF for single cell and bulk WGS. d) Phenotype prediction accuracy of different SNP sets under different sequencing depths.

Building on the advancements in GS for neonates, extending selection practices to the embryonic stage has emerged as a promising strategy, with GSE poised to significantly enhance the speed and efficiency of animal breeding programs.^[^ ^41^ ^]^ We hypothesized that DPImpute, which could infer genotype at genomic regions without NGS reads, might offer an accurate and cost‐effective approach to genotype SNPs in the context of GSE (**Figure** **5**a). To test this hypothesis, we collected a single blood cell from each of the three pigs of Duroc breed, and performed single‐cell genomic DNA library preparation followed by DPImpute‐based genotyping. The imputation panel is of the same breed with 2587 individuals to better represent the haplotypes present in the testing samples (Experimental Section). The genotyping concordance is 94.47% at a coverage of 2X (Figure 5b). Even at lower sequencing depth, such as 1X and 0.5X, the genotyping concordance remains accurate at 94.22% and 93.70%, respectively. Although decreasing sequencing coverage gradually decreased genomic concordance, single‐cell genotyping by DPImpute achieved a concordance rate greater than 88% at a sequencing coverage as low as 0.05X. Our previous results and other studies has suggested that genotyping accuracy at 85% is sufficient for genomic selection.^[^ ^42^ ^]^ Besides, for the same sample, single‐cell genotyping by DPImpute, compared to bulk genotyping by the same analytic framework, showed only a slight decrease at ultra‐low coverage sequencing (Figure 5c). These results demonstrate that DPImpute can achieve high‐accuracy genotyping for single‐cell low‐coverage data, comparable to bulk tissue samples.

To further validate the feasibility of genomic selection in animal embryos, we sequenced 12 single cells, each isolated from a pig blastocyst, followed by genotype imputation using DPImpute and the same Duroc imputation panel. This approach simulates a realistic scenario of genomic selection in animal embryos and provides compelling evidence for the application of DPImpute in such contexts. At 2X coverage, the genotyping concordance for blastocyst single cells was 94.89% (Figure 5b), a 0.42% improvement over single blood cell samples, and only a 3.09% reduction compared to NGS data from bulk tissue samples. Genotyping concordance for blastocyst single cells remained stable across most MAF regions when the sequencing coverage was ≥0.1X, closely aligning with the results from bulk tissue samples (Figure 5c). To assess the consistency of phenotypic predictions based on imputed SNPs from single blastocysts, we trained an rrBLUP model using all 1940 samples across seven real traits. For each of the 12 single cells at varying sequencing depths, we predicted phenotypic values for the seven traits. We then calculated the PCC scores for the predicted phenotypic values between pairs of single cells. Since these blastocysts originated from the same genetic donor, a high PCC indicates strong consistency in phenotype predictions across multiple runs of genetic testing. The results showed that the median PCC of phenotypic predictions exceeded 0.94 when the sequencing depth was ≥0.1X, with PCC values of 0.94, 0.96, 0.97, 0.99, 0.99, and 1.00 at sequencing depths of 0.1X, 0.2X, 0.3X, 0.5X, 1X, and 2X, respectively (Figure 5d). These findings underscore that using ultra‐low coverage sequencing followed by DPImpute genotyping is a viable approach for single‐cell genotyping in the context of genomic selection of animal embryos.

### 2.8 Web Server Development of DPImpute to Facilitate Its Usage

**Figure 6:**
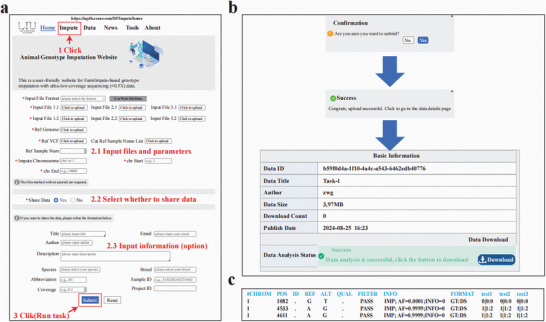
The usage of DPImpute platform on a web server. a) Overview of the platform's input functionality, illustrating the simple three‐step process for submitting jobs. First, users click the “Impute” button to access the imputation webpage. Next, users enter the required information into the platform. Finally, users complete the process by clicking the “Submit” button. b) Interface displaying the website's running progress and the results download option. c) Output generated by the DPImpute platform.

To promote the widespread adoption of DPImpute within the research community, we have developed a user‐friendly web server a graphical interface (https://agdb.ecenr.com/DPImpute/home). Upon accessing the website, users can initiate the DPImpute workflow in any web browser by clicking the “Impute” button in the navigation bar, which redirects them to the job submission webpage (**Figure** **6**a). Users can then upload their genotype input files, reference genome files, and reference dataset information. To enhance user convenience, the web server supports multiple input file formats, including FASTQ, FASTQ.GZ, BAM, and VCF.GZ, which can be selected through the “Input File Format” option (Figure 6a). After selecting the “Input File Format,” such as “fastq.gz,” users can click “Use Website Data” to analyze and test the functionality using existing data available on the website (Figure 6a). This feature enhances the convenience for users to explore and test the module's capabilities. To accommodate researchers working with various species, we allow users to upload their own reference genome files in FASTA format and different reference datasets.

To ensure data security and user privacy, all data uploaded by users will be deleted after the imputation jobs are completed. However, users have the option to share their data by selecting the “share data” option and can optionally provide additional data‐related information to benefit other users (Figure 6a). While providing an email address is optional, users can also name their jobs according to their preferences. Once the “Submit” button is clicked, the job information is submitted to the back‐end server (Figure 6a).

The output from DPImpute is a VCF.GZ file containing phased genotypes and genotype dosage. If users provide an email address when submitting their data, a download link for the genotyped results will be sent to their email. If no email address is provided, users can access a URL that will be displayed after clicking the “Submit” button to view and download their analysis results (Figure 6b). Upon completion of the program, users can click the “Download” button to retrieve the genotype imputation results. The results are stored in the standard VCF.GZ format, containing genotype information for each sample (Figure 6c). Additionally, users can browse and utilize data shared by others by clicking the “Data” button (Figure 6a).

To further ensure data security and user privacy, the analysis results are retained for three days before being automatically deleted, provided the “share data” option was not selected. For enhanced flexibility, a Docker container encompassing all pipeline components is also available, allowing users to perform imputation on their own servers with straightforward implementation (https://agdb.ecenr.com/DPImpute/home).

## 3 Discussion

In this study, we developed a computational framework, DPImpute, which demonstrated accurate genotype imputation in the context of small number of testing samples and limited size of reference panels. We showcased its capacity to increase the predication accuracy of GS by flexibly utilizing potential trait‐relevant SNPs in model training. Our findings also suggest that DPImpute holds substantial value for genomic selection in embryos, owing to its impressive imputation performance utilizing low‐coverage NGS data from single cells. To enhance accessibility, we have developed a user‐friendly web server and a Docker container for easy implementation. In summary, we believe that DPImpute not only possesses the potential to significantly expedite breeding programs by facilitating precise and cost‐effective genotyping, but also can serve as a valuable tool for genetics research on diverse populations, including both humans and animals.

In previous studies, BaseVar and BaseVar+STITCH were found to be effective for obtaining genotype information from a large number of low‐coverage sequencing samples. However, their genotype imputation accuracy diminishes significantly when the number of testing samples is small (Figure 2a). GATK and Beagle are widely used for genotype analysis in high‐depth sequencing data but demonstrate limited performance when applied to low‐coverage sequencing data. Methods like Beagle aim to impute all SNP sites in a single round, which is effective under conditions of minimal genotype missingness. However, in the context of ultra‐low‐coverage sequencing, characterized by extensive genotype missingness and limitations in both the number of testing samples and the size of reference panels, the applicability of Beagle is considerably restricted. While Minimac can be employed for low‐coverage genotype imputation, its accuracy in ultra‐low‐coverage scenarios still requires improvement (Figure 2a). Here, we show that DPImpute demonstrates superior genotype imputation performance under varying conditions, including different sequencing depths, sample sizes, reference panel sizes, and reference population types. This improvement becomes particularly evident after two rounds of imputation, where the complementary strengths of the tools used in each round enable DPImpute to outperform other imputation methods, especially in scenarios with small testing sample sizes, limited reference panel sizes, and ultra‐low sequencing depths. In the first round of imputation, we employed GLIMPSE due to its ability to accurately impute genotypes directly from the raw sequencing data of the testing samples. However, we observed that relying solely on GLIMPSE in this phase resulted in suboptimal overall genotype imputation accuracy, particularly under conditions of small testing sample sizes and ultra‐low sequencing coverage. This limitation arises from the absence of sequencing reads covering many SNPs, which hinders the successful imputation of their genotypes. To address this, we introduced a second round of imputation using IMPUTE2, a method adept at whole‐genome SNP imputation. By leveraging VCF files containing high‐confidence SNP genotypes from the first round, IMPUTE2 effectively filled in the remaining genotypes. This two‐step approach allowed the high‐quality partial genotypes generated by GLIMPSE in the first round to serve as a robust foundation for IMPUTE2, which subsequently expanded the imputation to cover the entire genome while maintaining accuracy. In essence, DPImpute integrates the strengths of GLIMPSE and IMPUTE2 within a streamlined framework, enhancing genotype imputation accuracy even in challenging scenarios, such as those involving small testing sample sizes and ulcWGS data.

To evaluate the computational cost of DPImpute, we also calculated the CPU hours and Random Access Memory (RAM) usage of DPImpute. We selected testing sample sizes of 1, 5, 10, 15, 30, 50, 100, and 200, all with a sequencing depth of 0.3X (Figure S8, Supporting Information). Each testing sample provided genotype information for 11633164 SNPs. We assessed the CPU hours and RAM required for these tests and derived a trend equation based on the results. According to this equation, performing genotype imputation for 1000 testing samples would require ≈2081.26 CPU hours and 579.81 GB of RAM (Figure S8, Supporting Information). Therefore, the estimated computational cost of imputing one sample is ≈0.04 dolloars, which is a minor proportion of the total cost of ulcWGS genotyping.

In the field of animal breeding, pre‐embryonic genome selection holds tremendous potential, particularly for species like cattle and sheep that usually give birth to a single offspring at a time. In this study, we simulated embryo genetic testing by applying DPImpute to genotype single cells derived from adult blood tissue. Additionally, we demonstrated the potential of DPImpute in more realistic scenarios, specifically in genotyping single cells sampled from blastocysts. To further enhance its applicability in GSE, several technical issues need to be addressed. For example, the turnaround time for DPImpute‐based genotyping must align with the precise timing of in vitro fertilization (IVF) and subsequent surrogate transplantation. Ensuring the efficiency and timeliness of genotyping is vital for the successful integration of DPImpute into the embryonic testing process. Additionally, given the diminishing role of sequencing cost due to extremely low sequencing depth, optimizing the expense of single‐cell WGS library preparation is paramount. In our present study, we employed MALBAC, a pricier choice compared to conventional WGS library preparation methods. It is imperative to explore cost‐effective alternatives while preserving WGS library quality, including genomic coverage uniformity. This is pivotal in making the technology financially viable and expediting its broad integration into animal breeding programs.

In addition to SNPs, many functional DNA variants involve alterations of more than one nucleotide, such as structural variations (SVs), which encompass a wide array of mutation types, including deletions, duplications (both tandem and interspersed), inversions and transpositions. Imputing SVs from low‐coverage sequencing remains challenging. Some research has attempted to impute small structural variations by converting them into SNP‐like encodings. Subsequently, they employed low‐coverage NGS data and genotype imputation software such as FImpute and Eagle2.3‐Minimac3 for imputation.^[^ ^43^ ^]^ Under this scenario, DPImpute provides an alternative approach to infer SVs from low‐coverage or ultra‐low coverage sequencing data. However, this approach typically yields only moderate results and struggles to accurately impute larger SVs. Furthermore, the gold standard and reference datasets for SVs are still in need of refinement.^[^ ^44^, ^45^ ^]^ This is primarily due to the prevalent use of short reads‐based NGS platforms in genotyping SVs within a large population.^[^ ^35^, ^46^ ^]^ While these platforms offer accuracy for smaller SVs, they lack precision in detecting larger SVs, resulting in reduced accuracy within repetitive regions.^[^ ^47^ ^]^ With longer sequencing reads, third‐generation sequencing platforms, such as the new PacBio HiFi and Oxford Nanopore Technologies, offer higher accuracy in detecting SVs. However, it is important to acknowledge that third‐generation sequencing comes at a higher cost compared to NGS, limiting its application to larger populations. Nevertheless, with the continuous decline in sequencing costs, the potential to conduct high‐depth third‐generation sequencing on larger populations is becoming more feasible. This progress holds promise for generating more precise and comprehensive reference panels for SV imputation.

Beyond genotype imputation from sparse sequencing data, recent advancements in imputation methods—particularly for single‐cell data analysis—have harnessed deep learning to overcome the challenges associated with sparse and multi‐modal datasets. For example, ImputeHiFI utilizes complementary structural information from single‐cell Hi‐C and RNA FISH data to achieve high‐fidelity spatial localization of chromatin loci, enhancing the resolution of cellular clustering, compartmentalization, and chromatin loop detection in mouse brain datasets.^[^ ^48^ ^]^ JAMIE employs a joint variational autoencoder to map multi‐modal projections into a shared latent space while preserving modality‐specific features, enabling the integration of diverse datasets such as gene expression and chromatin accessibility for mechanistic insights.^[^ ^49^ ^]^ Monae introduces a contrastive learning framework tailored for unpaired multi‐modal data, integrating regulatory relationships to enhance intra‐ and cross‐modal imputation, advancing tasks like cell‐type identification and gene regulatory network reconstruction.^[^ ^50^ ^]^ These innovative approaches underscore the potential of machine learning and deep learning in tackling sparse genomic datasets. Their success in single‐cell data integration and imputation suggests exciting possibilities for adopting similar methodologies in genotype imputation, enabling more comprehensive insights into genetic architecture and functional genomics.

## 4 Experimental Section

### Ethics Statement

All experimental procedures associated with the pigs used in this study were conducted following guidelines established by the Ministry of Science and Technology (Beijing, China). Ethical approval(AGIS‐ER‐2024‐002) was granted by the Ethics Committee of Life Sciences, Agricultural Genomics Institute, Chinese Academy of Agricultural Sciences(Shenzhen, China).

### Low Coverage Whole‐Genome Sequencing Data Preprocessing

The read quality of raw sequencing fastq data was assessed and using fastp,^[^ ^51^ ^]^ then, adapters and low‐quality bases were removed by fastp.^[^ ^51^ ^]^ After that, sample reads were mapped to the reference genome using BWA.^[^ ^52^ ^]^ Notably, DPImpute could automatically invoke Fastp and BWA to perform these tasks. For details about the software versions and parameter Settings mentioned above, see Table S3 (Supporting Information).

### SNP Calling by DPImpute

DPImpute employs a two‐round imputation strategy designed to achieve accurate SNP imputation. In the first round, GLIMPSE(v1.1.0) was used to construct haplotypes by integrating both the reference panel data and ultra‐low coverage whole‐genome sequencing (ulcWGS) data from the testing samples.^[^ ^21^ ^]^ This was achieved by setting the parameters “GLIMPSE_chunk –window‐size 2 000 000 –buffer‐size 200 000′ and ‘GLIMPSE_phase –reference” to optimize haplotype phasing and imputation. These newly generated haplotypes were leveraged to impute SNPs for the testing samples by applying the default parameters “GLIMPSE_ligate” and “GLIMPSE_sample –solve”. Additionally, this initial imputation round converts the alignment files of the testing samples into VCF format, which serves as input for the second imputation round.

In this subsequent round, the reference panel undergoes self‐imputation using Beagle(v5.1) to infer the missing SNPs with the parameters “java ‐Xmx152212 m ‐Xms132212 m ‐Xmn132212 m ‐XX:‐UseGCOverheadLimit nthreads = 5′.^[^ ^22^ ^]^ The self‐imputed reference panel was then used to impute the vcf file of the testing samples generated from the first round, using IMPUTE2(v1.1.4). IMPUTE2 leverages the positional Burrows‐Wheeler transform (PBWT), an efficient method for managing genetic data, to carry out the final imputation by applying the default parameters ‘imp5Converter” and “impute5”. To enhance the accessibility of DPImpute, a user‐friendly web server (https://agdb.ecenr.com/DPImpute/home) was developed and a Docker container for seamless implementation. The installation and usage of DPImpute can be viewed on the website(https://agdb.ecenr.com/DPImpute/tool).

### SNP Calling by Other Tools

A range of widely used tools were evaluated for SNP calling and Imputation. These tools include GATK,^[^ ^53^ ^]^ BaseVar,^[^ ^28^ ^]^ BaseVar+Stitch,^[^ ^27^, ^33^ ^]^ and Minimac.^[^ ^54^ ^]^ Tools were applied with their “default” options, albeit with a few exceptions. The software version and parameter Settings is detailed in Table S3 (Supporting Information).

### Evaluation of Imputation Accuracy

To assess the accuracy of DPImpute under different parameters for SNP imputation of ultra‐low coverage sequencing data, a published dataset consisting of 2802 Duroc boars was exploited,^[^ ^33^ ^]^ with an average sequencing depth of 0.73X. The genotyping results of this dataset demonstrated a concordance rate of 97.0% compared to high‐coverage sequencing data and 99.0% when compared to microarray data, making it a reliable reference to serve as the “ground truth” for genotyping accuracy evaluation and as the reference panel for imputation.

By comparing the imputed genotypes with the true genotypes, genotype concordance was calculated and r^2^. Genotype concordance was calculated as the number of concordant genotype sites between imputed low‐coverage genotypes and true genotypes, divided by the total number of all true genotype sites. The calculation steps of r^2^ were as follows: First, compute the covariance between the imputed genotypes (X) and the true genotypes (Y), denoted as Cov(X,Y). Then, calculate the standard deviations of the imputed genotypes and the true genotypes (σ(X) and σ(Y)). The formula for r^2^ is:

(1) $$r^{2}={\left(\mathrm{Cov}\left(X,Y\right)\right)}^{2}/\left(Var\left(X\right)\cdot Var\left(X\right)\right)$$

where Var(X) and Var(Y) represent the variance of the imputed and true genotypes, respectively. The mean genotypic r‐squared was calculated for the different genotype sites called by each method. Concordance and r‐squared were further evaluated across different minor allele frequency (MAF) bins to examine DPImpute's performance across SNPs with varying MAFs.

In each iteration, 200 testing samples were randomly selected from these 2802 Duroc boars,^[^ ^33^ ^]^ and performed SNP imputation at different sequencing depths, ranging from 0.001x to 0.5x. Given only 31 samples with sequencing depths exceeding 1x, higher coverage scenarios were simulated by randomly selecting 15 samples with sequencing coverage greater than 10x and generating data at coverages from 1x to 10x. Additionally, a separate dataset containing 3202 high‐depth sequencing samples was utilized.^[^ ^35^ ^]^ From this dataset, 30 testing samples representing five ethnic populations were randomly selected and subjected to the same strategy to simulate sequencing coverages ranging from 0.001x to 10x.

To comprehensively evaluate the performance of DPImpute across various testing sample sizes, an assessment of imputation accuracy was conducted using a diverse range of sample sizes with a sequencing depth of 0.3X, spanning from 1 to 200. For a thorough comparison, 20 repetitions were conducted considering inter‐sample variations when the sample size was 1. As the sample size increased from 5 to 100, 5 repetitions were carried out for each increment. When dealing with the ample sample size of 200, a single repetition sufficed.

To conduct a comparison of imputation accuracy using reference panels of different sizes, the size of testing samples was kept fixed at five and varied the size of reference panels from 100 to 2000. The imputation accuracy was then averaged from five separate analyses, ensuring reliable and the calculation of statistical significance.

To evaluate the robustness of DPImpute, a multi‐population dataset was utilized comprising 3202 individuals from the 1000 Genomes Project.^[^ ^35^ ^]^ This dataset includes high‐accuracy genotype information derived from high‐depth sequencing, serving as a gold standard for assessing genotype imputation accuracy. Five ethnic groups—PUERTO RICAN in Puerto Rico (PUR), PUNJABI in Lahore, Pakistan (PJL), GAMBIAN in Western Division, The Gambia (GWD), HAN Chinese from southern China (CHS), and CEPH/UTAH Pedigree (CEU)—were selected. From each group, 6 samples were randomly chosen for testing (Figure 2h,i). A reference panel was constructed using the remaining 3000 individuals, and genotype imputation for the 30 testing samples was performed using this reference panel.

### Phenotype Simulation

In the simulation process, the phenotypic values (P) of each individual (*i*) were generated using the following equation:

(2) $$P_{i}=\mu +s\mathrm{TB}V_{i}+e_{i}$$

Here, µ represents the overall mean, *s*TBV_*i*_ represents the true breeding value calculated as the sum of QTL (Quantitative Trait Locus) effects multiplied by genotypes. The effects of QTL were sampled from a gamma distribution with parameters gamma(0.4, 1.66) (Hayes and Goddard, 2001). The sign of the QTL effect was assumed to be either positive or negative with a probability of 0.5.

The term *e_i_* represents random error for each sample *i*, assumed to follow a normal distribution N(0, $\sigma_{e^{2}}$). The residual variance $\sigma_{e^{2}}$ is a constant value, and the genetic variance ($\sigma_{g^{2}}$) at different heritability levels is determined by $\sigma_{g^{2}}=\sigma_{e^{2}}\;*h^{2}/\left(1-h^{2}\right),$ where *h* ^2 ^represents the heritability. After calculating the genetic variance, the standardized true breeding values (*s*TBV_*i*_) were produced by multiplying the TBVs by the square root of $\sigma_{g^{2}}$ divided by the variance of TBVs.

This simulation approach allows for the generation of phenotypic values that incorporate genetic effects (through QTL) and random error, with the heritability controlling the proportion of phenotypic variation attributed to genetic factors. The standardized true breeding values provide a normalized representation of the genetic effects, enabling further analysis and evaluation of the breeding values in the simulation.

### GS Modeling and Phenotype Prediction

In this study, rrBLUP model was used to predict phenotypes from each set of SNPs, which is one of the most extensively used and robust regression models for phenotype prediction.^[^ ^55^ ^]^ rrBLUP assumed the effect values of SNPs follow a normal distribution with mean 0, and a small but non‐zero variance by the following equation:

(3) Y=μ+Zg+e

Here, *Y* represents the phenotypes of all samples, µ represents the overall mean, *Z* represents the genotype matrix, $g∼N(0,I\sigma_{g}^{2})$ represents the marker effects, $I\sigma_{g}^{2}$ represents the small but none zero variance, *N*(■) represents the normal distribution, *e* represents the residual effects.

When attempting to solve the model using the “mixed.solve” function in the R package “rrBLUP”,^[^ ^56^ ^]^ difficulties were encountered due to the large size of the genotype matrix (tens of millions of SNPs). The “mixed.solve” function allows solving the model using an alternative parameter, K, which represents the kinship matrix. However, in the “rrBLUP” package, the kinship matrix K was typically calculated using the “A.mat” function, which derives K from the genotype matrix. Given the size of the dataset, direct computation of K using “A.mat” was not feasible due to excessive memory requirements. To overcome this challenge, the GCTA^[^ ^57^ ^]^ software was utilized to calculate the kinship matrix among samples using the parameters “–make‐grm –make‐grm‐alg 1′. This approach significantly reduced memory usage and sped up the computation. Finally, the rrBLUP model was implemented using the ‘mixed.solve” function in the rrBLUP package with the pre‐calculated kinship matrix K.

### Jaccard Index Calculation

In this study, Jaccard index (JI) was used to evaluated the consistence of phenotype prediction. For each simulated trait (i), Y_i_ denotes the sorted predicted phenotypes in order from greatest to smallest. For each round (j) of imputation, the top‐ranked samples’ set A_i,j, α_ at ratio α% were defined using the following equation:

(4) $$A_{i,j,\alpha }=\left[Y_{i,j,1},Y_{i,j,2},\text{…},Y_{i,j,\beta }\right]$$

Here, β  =  ceiling(200*α/100), ceiling(·) denotes the R function that returns the smallest integer not less than the corresponding element. Finally, for each simulated trait (i), for each ratio α%, the JI_k,h_ between different two rounds k and h was calculated using the following equation:

(5) $$JI_{k,h}=N_{A_{i,k,\alpha }\cap A_{i,h,\alpha }}/N_{A_{i,k,\alpha }\cup A_{i,h,\alpha }}$$

Here, $N_{A_{i,k,\alpha }\cap A_{i,h,\alpha }}$ represents the samples recognized as top‐ranked α% by both of the two rounds, $N_{A_{i,k,\alpha }\cup A_{i,h,\alpha }}$ represents the samples recognized as top‐ranked α% by either one of the two rounds.

### Flexible SNP Selection in GS Modeling and Trait Prediction

To select potentially functional SNPs from GWAS significant hits, genome‐wide complex trait analysis (GCTA, version 1.94.0) software^[^ ^57^ ^]^ was used to conduct a GWAS on 10 randomly selected simulated traits, each with a heritability of 0.7 and 100 QTLs, across 2802 individuals, as well as on 7 real traits. SNPs with a P‐value < 1.0e^−12^ were considered as potential functional candidates.

Genomic locations were gathered from the GeneSeek porcine 50K SNP chips, originally based on the Sus Scrofa 10.2 assembly. To update these locations to the Sus Scrofa 11.3 assembly, the liftOver tool was used from the UCSC Genome Browser (http://genome.ucsc.edu/cgi‐bin/hgLiftOver). These positions were then extracted from the full genotype dataset using BCFtools^[^ ^58^ ^]^ to create a subset containing only the array loci.

To explore potential cis‐regulatory variants in pig skeletal muscle that could predict lean meat deposition (LMD), the DeepSATA model was employed to analyze ATAC‐Seq data from pig skeletal muscle (accession number GSE143288).^[^ ^59^ ^]^ The model quantified and ranked the impact of each SNP, imputed using DPImpute, on cis‐regulatory activity. From this analysis, the top 32451 SNPs (the same number as on the array) was selected for further study.

### Single Blood Cell Sample Collection

A single blood cell was collected from each of the three pigs of Duroc breed for both single‐cell sequencing and whole‐genome sequencing. To obtain the genomic DNA necessary for these analyses, ear tissue samples were collected from the selected pigs and subjected to extraction using the DNeasy Blood & Tissue Kit (Qiagen 69 506).

### Single Blastocyst Cell Collection

To obtain single blastomeres from blastocysts, handmade cloning was performed as previously described.^[^ ^60^ ^]^ Briefly, porcine ovaries were collected from prepubertal gilts (Duroc × Landrace × Large White) at a local slaughterhouse. Cumulus‐oocyte complexes (COCs) were aspirated from 3–6 mm ovarian follicles using a 10 ml syringe with an 18‐gauge needle. COCs with multiple layers of intact cumulus cells were selected and cultured in the in vitro maturation (IVM) medium^[^ ^61^ ^]^ at 38.5 °C in a 5% CO_2_ incubator for 42–44 h. The COCs were then treated with 1 mg ml^−1^ hyaluronidase to remove the cumulus cells. To enucleate the oocytes, a third of the cytoplasm near the polar body was excised using a sharp blade after partial digestion of the zona pellucida with 3 mg ml^−1^ pronase.

Porcine fetal fibroblasts were isolated from male Duroc fetuses at 7 days gestation to serve as donor cells. Each cytoplast was fused with a single donor cell via an electric pulse of 2.0 Kv cm^−1^ for 9 µs. After 1 h, the fused cytoplast‐donor cell pairs were fused with an additional cytoplast and activated with a second electric pulse of 0.86 kV cm^−1^ for 80 µs. Following chemical activation with 5 mg ml^−1^ cytochalasin B and 10 mg ml^−1^ cycloheximide for 4 h at 38.5 °C in an incubator with 5% CO_2_ and 5% O_2_, the cloned embryos were cultured in porcine zygote medium‐3 (PZM‐3)^[^ ^62^ ^]^ using the Well of the Well (WOW) system^[^ ^63^ ^]^ for 7 days to obtain blastocysts.

The blastocysts were washed three times in phosphate‐buffered saline (PBS) containing 0.1% polyvinyl alcohol (PVA), and then placed in 0.25% trypsin for 10–20 min at 37 °C. Single blastomeres were isolated by repeated pipetting with a finely pulled glass tip, washed three times in 0.1% PVA/DPBS, transferred into lysis buffer in a 200 µL eppendorf tube, and immediately frozen in liquid nitrogen.

### Single Cell Sequencing

The process of selecting single cells for sequencing involves several steps. First, the cells were visually inspected under a microscope to identify those with complete cell morphology and good activity. These selected cells were then carefully aspirated using an oral pipette. It was important to handle the cells delicately during this process. After aspirating the cells from the initial suspension, they were washed in droplets of a PBS‐BSA solution. This step helps remove any contaminants and prepares the cells for further processing. The washed cells were then transferred to a specialized lysate, specifically the Malbac lysate provided by the company. During this transfer, it was crucial to minimize the introduction of excess buffer, ensuring that no more than 0.5 µl of buffer was added. Once the single cells were placed in the lysate, a slight centrifugation step was performed at 4 °C. This helps ensure proper mixing and distribution of the cells within the lysate. After centrifugation, the lysate containing the single cells was stored at ‐80 °C. This storage temperature helps maintain the integrity of the cells and their genetic material until further processing. When transporting the samples, it was important to take precautions to protect the integrity of the samples. The pipettes containing the lysate and cells were placed in appropriate tube boxes and the box lid was securely covered and sealed. The tube box was then placed in a container with dry ice to maintain a low temperature and prevent any damage to the samples during transportation.

During the single‐cell genome sequencing sample collection process, the Malbac method was employed for the samples in the single cell collection tube. The resulting amplified product exhibited a length range of 300–2000 base pairs. To ensure the quality of the amplified products, purification and recovery were performed using Beckman Ampure XP magnetic beads. The concentration of the amplification products was measured using the Qubit^3.0^ Fluorometer from Life Technologies, CA, USA. Furthermore, the distribution of fragments in the amplified product samples was analyzed using Fragment Analyzer 1.0.2.9. Based on the test results, an assessment of the amplification product quality was made. An amplification test report was generated and provided to the customer for feedback on whether to proceed with library construction. This feedback allows for a collaborative decision‐making process, ensuring that the subsequent steps in the sequencing workflow align with the customer's requirements and expectations.

For library construction, a starting template of 5 ng of the amplified product from each sample was utilized. The Tn5 digestion method was employed to fragment the sample DNA into small, appropriately sized fragments. The fragmented samples then underwent several steps, including end repair, addition of base A, and addition of a linker. Following each reaction step, purification was performed using Beckman Ampure XP magnetic beads. The resulting spliced products were subjected to PCR amplification, with unique Index tags introduced into each sample. These Index tags served to distinguish the samples from one another during the sequencing process. The PCR amplification products were subsequently purified using Beckman Ampure XP magnetic beads to recover a library of fragments with the desired size. This final pre‐sequencing library was prepared for further analysis.

After the library construction was completed, the Fragment Analyzer 1.0.2.9 was utilized to evaluate the fragment length distribution of the library. Once the library met the expected standards, the effective concentration of the library was accurately quantified using quantitative polymerase chain reaction (Q‐PCR). To ensure high‐quality library preparation, the effective concentration of the library needed to exceed 10 nmol L^−1^. Libraries that passed the quality assessment were subjected to further testing, and the PE150 double‐ended sequencing program was performed using the Novaseq sequencing platform.

### Bulk Sample WGS Library Generation and Sequencing

The DNA was first fragmented using a restriction enzyme and incubated at 55 °C for 10 min. Purification of the fragmented DNA was performed using Backman AMPure XP magnetic beads. For PCR enrichment, the following steps were carried out: a hot cap step at 105 °C, followed by incubation at 72 °C for 3 min, a 30 s denaturation step at 98 °C, and 5–15 cycles consisting of a 15 s denaturation step at 98 °C, a 30 s annealing step at 60 °C, and an extension step at 72 °C for 3 min. The PCR reaction was finalized with an extension step at 72 °C for 5 min, followed by a hold at 4 °C. The resulting PCR products were purified using AMPure XP magnetic beads, which also sorted the fragments. To assess the quality and size of the library, Fragment Analyzer 1.0.2.9 was employed, revealing that the majority of fragments fell within the range of 300–700 bp. For accurate quantification of the library's effective concentration, the Qubit system was utilized. It was crucial for the effective concentration of the library to exceed 10 nmol L^−1^ to ensure high quality. Subsequently, the library was subjected to on‐machine sequencing using the Illumina Novaseq technology sequencing platform. The Paired End sequencing (PE150) method was employed for the sequencing process. These procedures ensured the availability of high‐quality DNA samples for subsequent genetic analyses and imputation using DPImpute.

### Data Availability

The single cell sequencing raw data in this study were deposited into NCBI and could be accessed via accession No. PRJNA1026661 and deposited in the Genome Sequence Archive in National Genomics Data Center, China National Center for Bioinformation / Beijing Institute of Genomics, Chinese Academy of Sciences^[^ ^64^ ^]^ (GSA: CRA012950) that are publicly accessible at https://ngdc.cncb.ac.cn/gsa. The sequencing raw data of single pig breed population in this study were downloaded from NCBI and can be accessed via accession Nos. PRJNA681437 and PRJNA712489. The sequencing raw data of multi‐ancestry human populations in this study were downloaded from 1000 Genomes Project by NCBI and could be accessed via accession Nos. PRJEB31736, PRJNA59833 and PRJEB36890.

## Conflict of Interest

The authors declare no conflict of interest.

## Author Contributions

W.Z. and W.M. contributed equally to this work. Y.L. and W.G.Z. designed and supervised the project. W.G.Z. designed the framework of DPImpute and performed genotype imputation analysis. W.M. performed genomic selection analysis. Z.C. obtained single blastomeres from blastocysts. C.W., T.S., W.D., S.Z, and W.J.Z. download data and processed phenotypic data. W.G.Z. and W.M. wrote the manuscript. Y.L., Y.Z., K.L., and Z.T. improved the manuscript. All authors read and approved the final manuscript.

## Supporting information



Supporting Information

## Data Availability

The data that support the findings of this study are openly available in NCBI at https://dataview.ncbi.nlm.nih.gov/object/PRJNA1026661?reviewer=9hd9jsdnd2i2urqj3evp65sjj, reference number 62.
