# Supplementary material for: DPImpute: A Genotype Imputation Framework for Ultra‐Low Coverage Whole‐Genome Sequencing and its Application in Genomic Selection
Source: Adv Sci (Weinh). 2025 Feb 27;12(16):2412482. doi: 10.1002/advs.202412482 (PMC12021046; doi:10.1002/advs.202412482)
Supplement: Supplementary file 1 — Supporting Information [file ADVS-12-2412482-s001.docx]

**
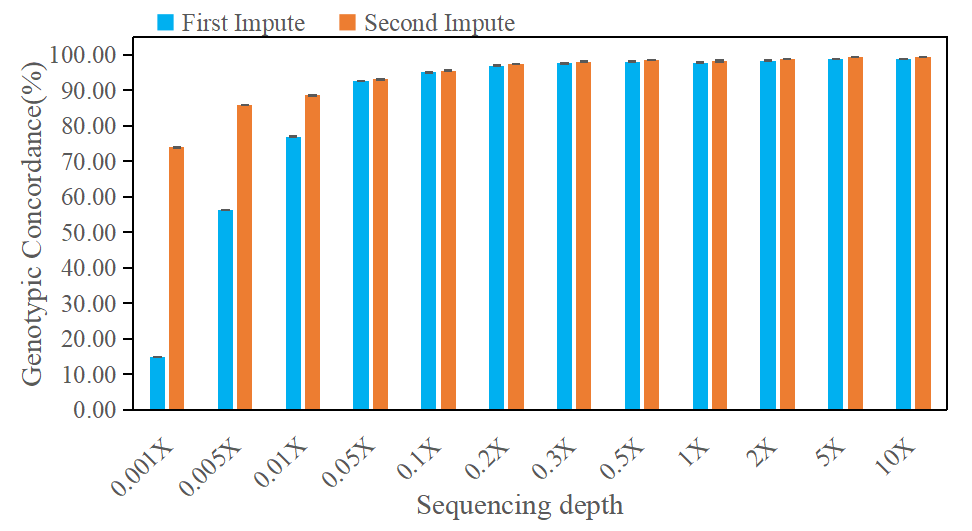
**

**Supplementary Fig. 1 |** **Comparison of imputation performance under different sequencing depths for single breed pig population.** All tests employed a reference panel consisting of high-accuracy genotype data of 2,587 individuals. Testing samples are distinct and do not overlap with the reference samples. For sequencing depths between 0.001X and 0.5X, 200 samples were used as testing samples. Due to limited sequencing capacity, testing sample size was reduced to 15 for sequencing depths ranging from 1X to 10X. 'First impute' represents the genotypic imputation accuracy following the initial round of genotype imputation in DPImpute, while 'Second impute' indicates the accuracy after the second round of imputation


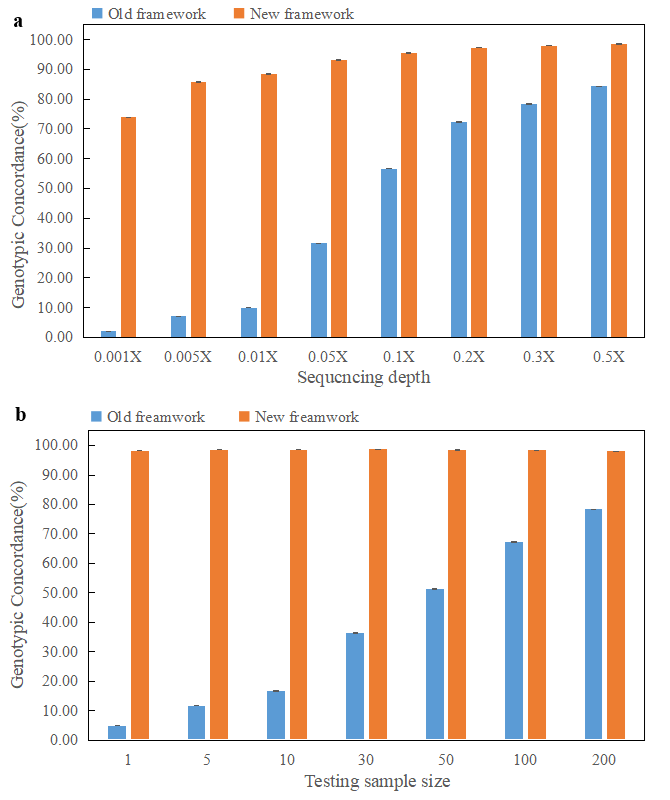


**Supplementary Fig. 2 | Genotype imputation performance was compared with different frameworks.**

(a) Comparison of genotype imputation performance across different frameworks and sequencing depths. All tests employed a reference panel consisting of high-accuracy genotype data of 2,587 individuals. Testing samples are distinct and do not overlap with the reference samples. For sequencing depths ranging from 0.001X to 0.5X, 200 samples were used as testing samples. The old framework employed the BaseVar algorithm to identify polymorphic sites and infer allele frequencies, followed by STITCH for SNP imputation. The new framework, DPImpute, utilized the GLIMPSE algorithm to identify polymorphic sites and infer allele frequencies and applied both GLIMPSE and IMPUTE2 for SNP imputation. (b) Comparison of genotype imputation performance across different frameworks and testing sample sizes. All tests utilized high-accuracy genotype information from 2,587 reference samples, with a sequencing depth of 0.3X for all testing samples. The old framework used the BaseVar algorithm to identify polymorphic sites and infer allele frequencies, followed by STITCH for SNP imputation. In contrast, the new framework, DPImpute, applied the GLIMPSE algorithm for polymorphic site identification and allele frequency inference, followed by SNP imputation using both GLIMPSE and IMPUTE2.

**
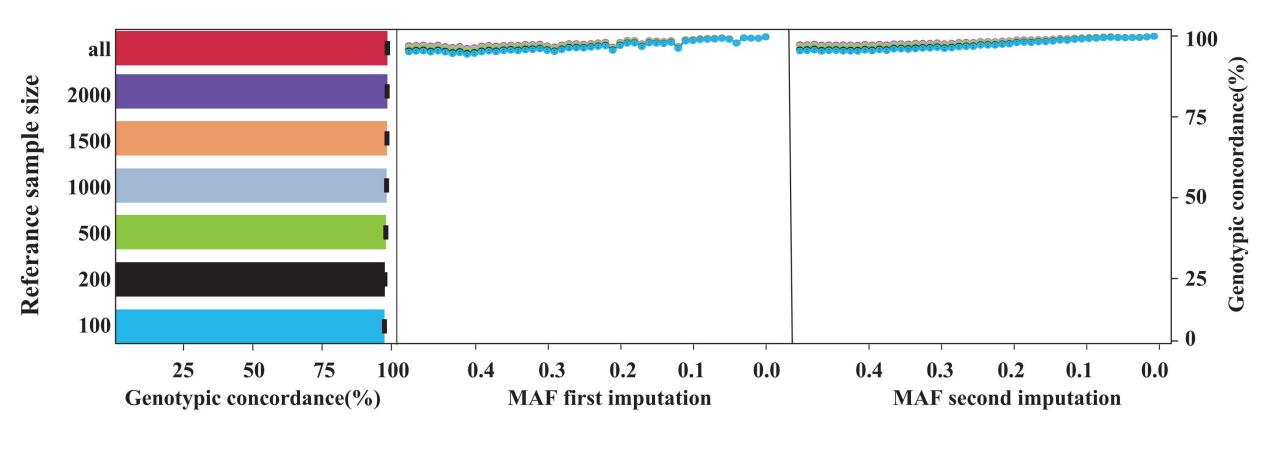
**

**Supplementary Fig. 3 | Comparison of imputation performance under different reference sample sizes.** Genotype imputation performance was evaluated across different reference sample sizes, while maintaining a consistent sequencing depth of 0.3X for all testing samples. The total number of testing samples for each test was set at 200. All tests utilized high-accuracy genotype information from 2587 reference samples as the reference.

**
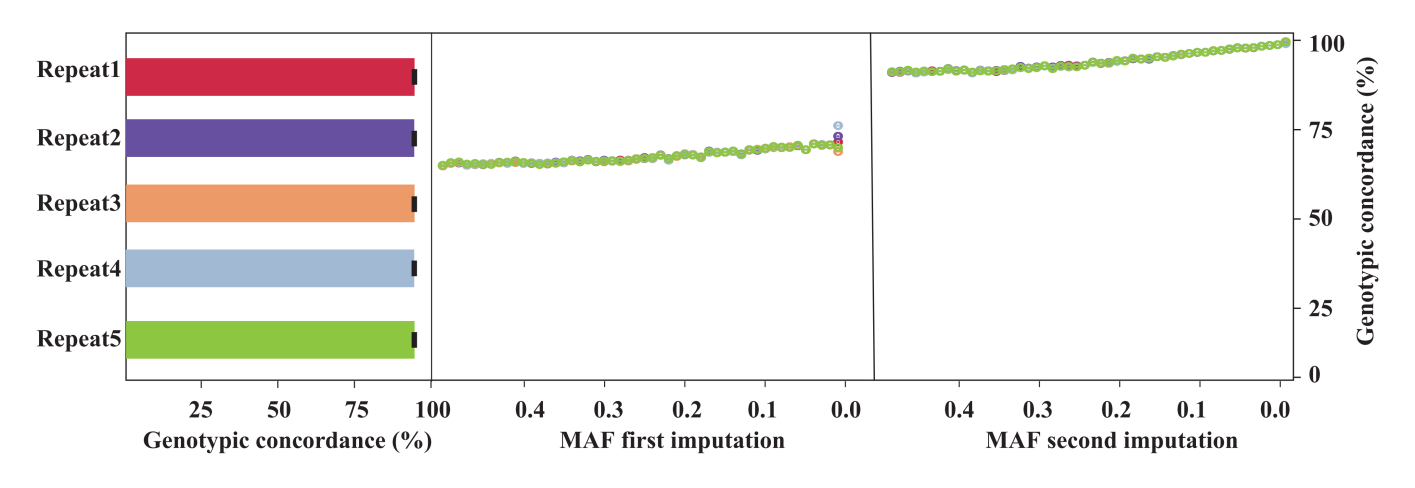
**

**Supplementary Fig. 4 | Imputation stability analysis of DPImpute.** Each repeat utilized high-accuracy genotype information from 100 reference samples as the reference. 10 samples not included in the 100 referrence samples were used as testing samples, down-sampled to 0.1X five times, followed by DPImpute analysis.


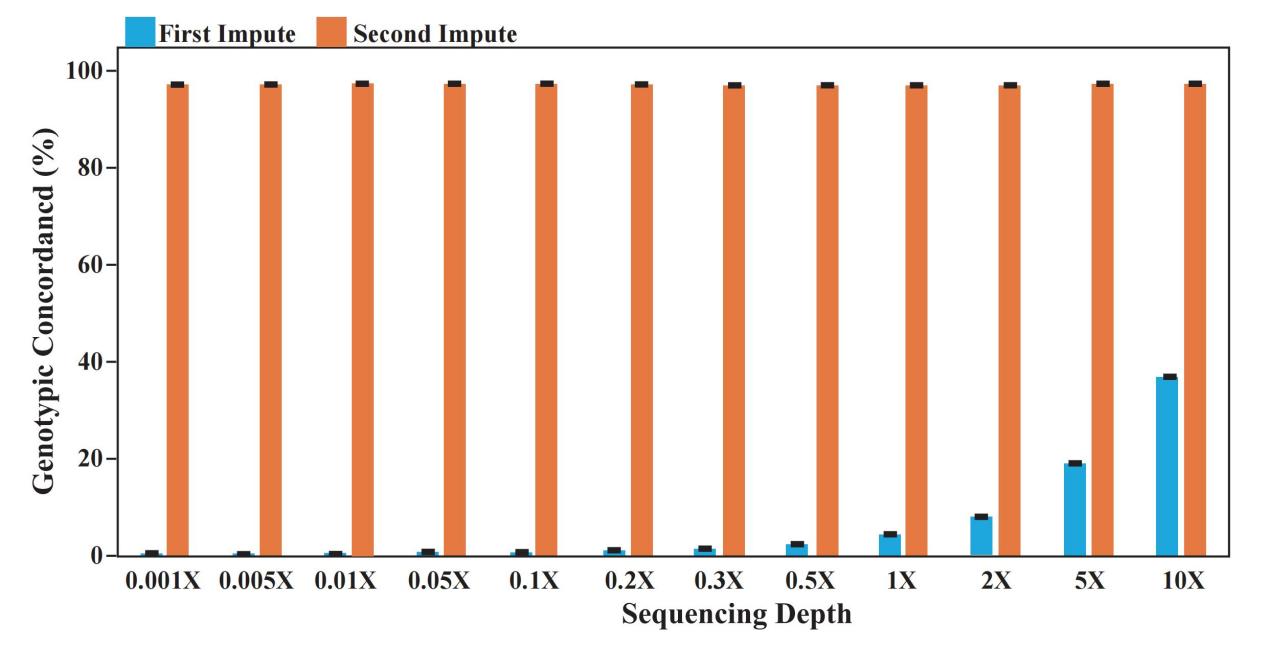


**Supplementary Fig. 5 | Imputation performance was compared with different sequencing depth for multiple human population.** In all tests, high-accuracy genotype information from a reference panel of 3,000 samples was used, with no overlap between testing and reference samples. The testing sample size was consistently maintained at 30 individuals across sequencing depths ranging from 0.001X to 10X. 'First impute' refers to the genotype imputation accuracy results after the first round of imputation using DPImpute, while 'Second impute' represents the accuracy results following the second round of imputation.


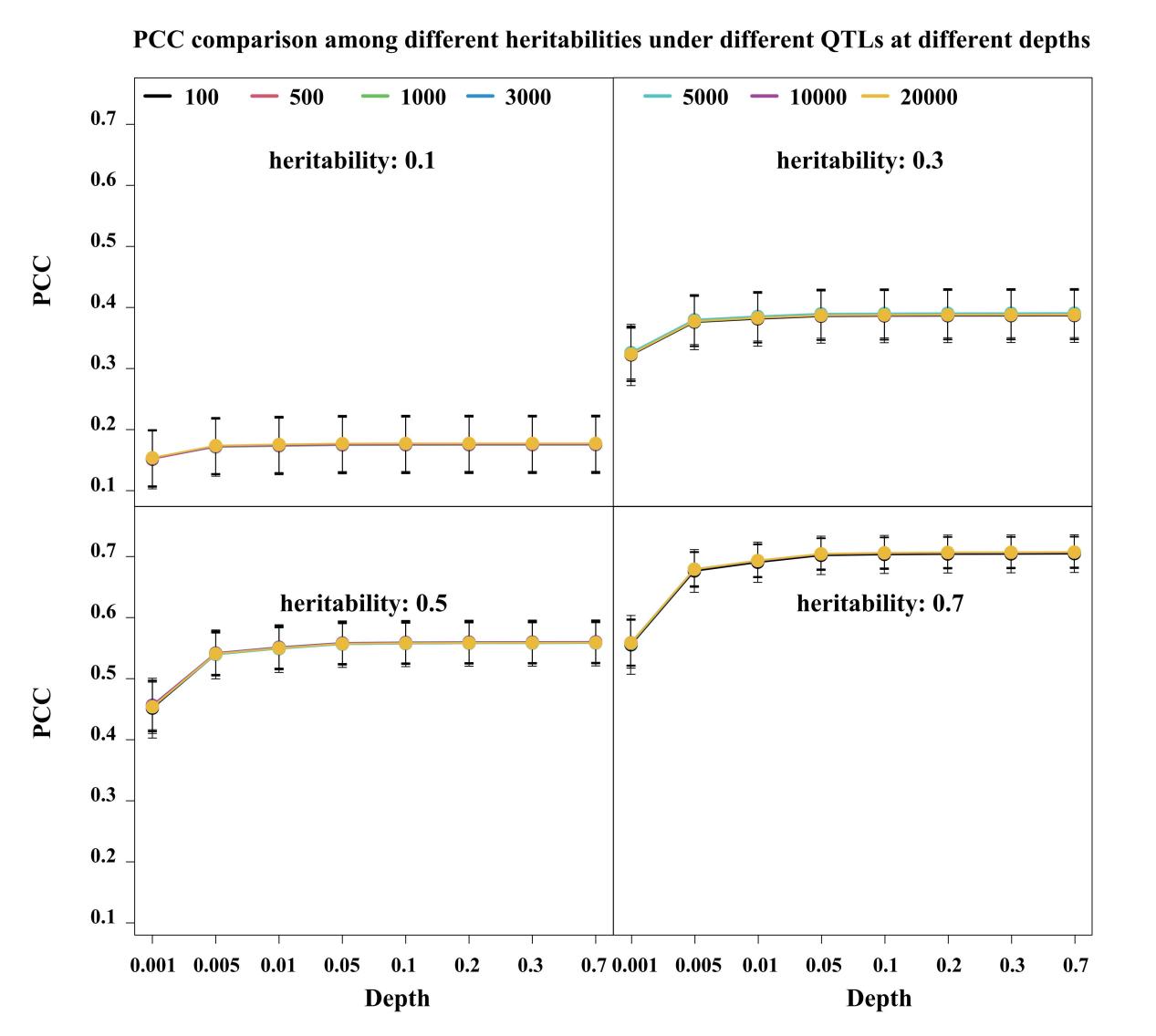


**Supplementary Fig. 6 | Phenotype prediction on simulated data among different heritability under different number of QTLs.** For each simulation scenario involving different combinations of heritability and QTL, we simulated 2,000 traits. The rrBLUP model was trained using 2,302 samples with a sequencing depth of 0.7X and subsequently used to predict the phenotypes of an additional 500 test samples, which exhibited sequencing depths ranging from 0.7X to 0.001X. Each dot in the figure represents the average PCC of the 2,000 simulated traits for each scenario.

**
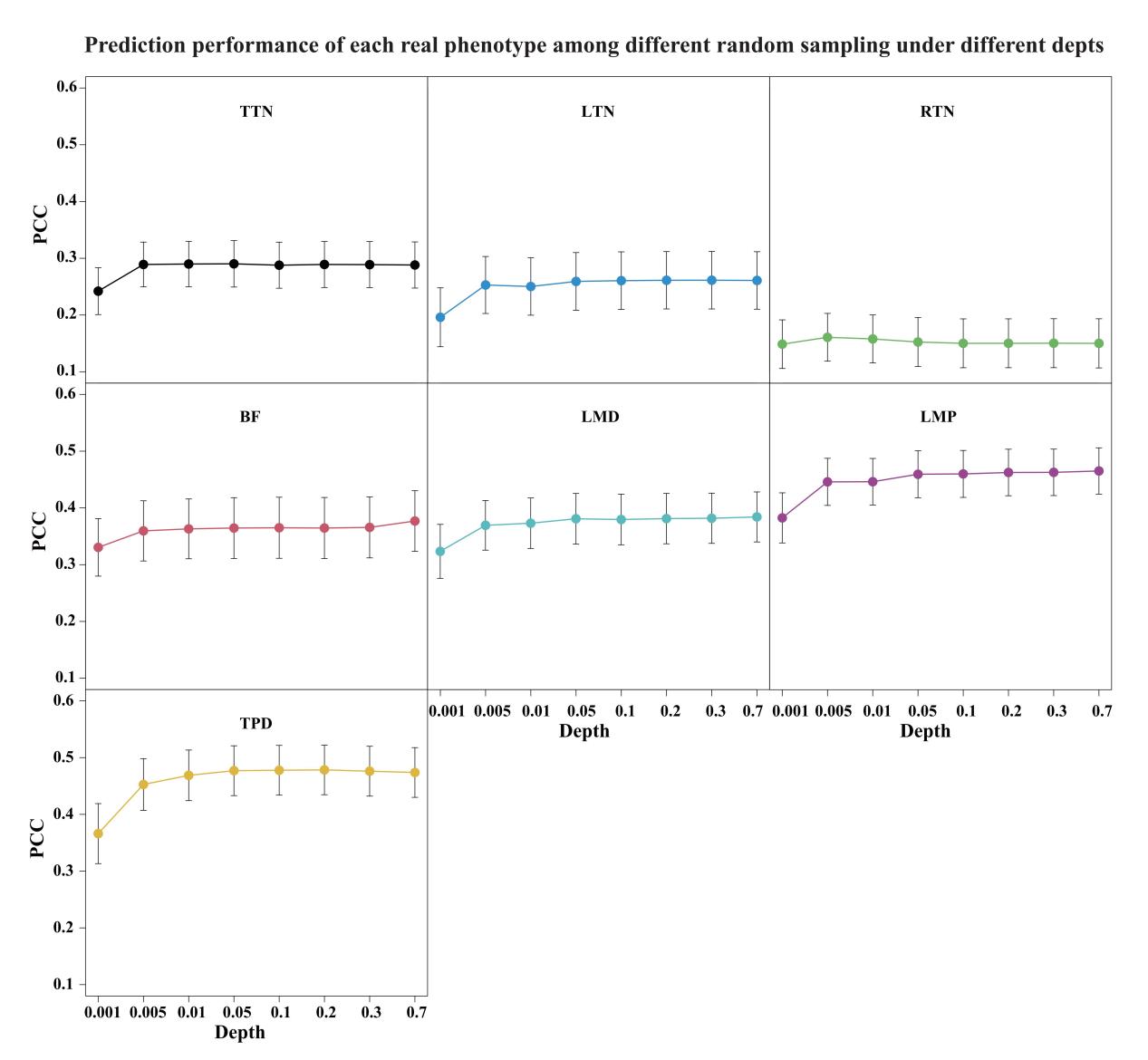
**

**Supplementary Fig. 7 | Phenotype prediction on real data under different depts.** For each real trait, the rrBLUP model was trained on 1,000 randomly selected samples with a sequencing depth of 0.7X and used to predict the phenotypes of 200 randomly selected testing samples with sequencing depths ranging from 0.7X to 0.001X. This process was repeated 100 times. Each dot in the figure represents the average PCC between predicted and real phenotypes across the 100 replicates for each real trait.


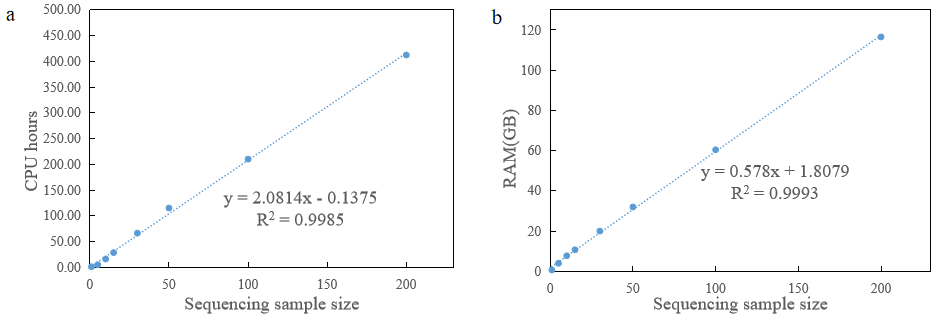


**Supplementary Fig. 8 | The CPU time and memoery of DPImpute across different testing sample sizes.** (a) The CPU hours of DPImpute across different testing sample sizes. (b) The Random Access Memory (RAM) of DPImpute across different testing sample sizes.

**Supplementary Table 1. Comparison of imputation performance under different sequencing depths for a single pig population.** All tests employed a reference panel consisting of high-accuracy genotype data of 2,587 individuals. For tests with sequencing depths ranging from 0.001X to 0.5X, 200 samples were utilized as testing samples. However, due to limited sequencing capacity, only 15 samples were used for tests with sequencing depths ranging from 1X to 10X. The term 'First Impute' refers to the accuracy of genotype imputation following the initial ground genotype imputation in DPImpute. 'Second Impute' indicates the accuracy of genotype imputation after the second round of ground genotype imputation.

| Sequencing Depth | First Impute | Standard error | Second Impute | Standard error |
| --- | --- | --- | --- | --- |
| 0.001X | 14.79 | 0.0174 | 73.89 | 0.0972 |
| 0.005X | 56.25 | 0.0781 | 85.83 | 0.1213 |
| 0.01X | 76.94 | 0.1048 | 88.58 | 0.1211 |
| 0.05X | 92.60 | 0.1160 | 93.09 | 0.1163 |
| 0.1X | 95.02 | 0.1066 | 95.50 | 0.1069 |
| 0.2X | 96.87 | 0.0919 | 97.36 | 0.0922 |
| 0.3X | 97.53 | 0.0821 | 98.03 | 0.0824 |
| 0.5X | 98.07 | 0.0691 | 98.57 | 0.0693 |
| 1X | 97.74 | 0.1913 | 98.24 | 0.1923 |
| 2X | 98.29 | 0.1350 | 98.79 | 0.1360 |
| 5X | 98.79 | 0.0662 | 99.29 | 0.0667 |
| 10X | 98.89 | 0.0371 | 99.39 | 0.0374 |

**Supplementary Table 2. Genotype imputation performance was compared across different frameworks.**

(a) Genotype imputation effect was compared with different freamwork and sequencing depth. All tests employed a reference panel consisting of high-accuracy genotype data of 2,587 individuals. For sequencing depths ranging from 0.001X to 0.5X, 200 samples were used. The old framework employed the BaseVar algorithm to identify polymorphic sites and infer allele frequencies, using STITCH for SNP imputation. In contrast, the new framework, DPImpute, utilized the GLIMPSE algorithm for identifying polymorphic sites and inferring allele frequencies, along with GLIMPSE and IMPUTE2 for SNP imputation. (b) The impact of genotype imputation was assessed with varying frameworks and sample sizes. All tests employed a reference panel consisting of high-accuracy genotype data of 2,587 individuals, with the sequencing depth for all testing samples set at 0.3X. Again, the old framework used the BaseVar algorithm for polymorphic site identification and allele frequency inference, as well as STITCH for SNP imputation, while DPImpute applied the GLIMPSE algorithm and both GLIMPSE and IMPUTE2 for SNP imputation

a

| Sequencing Depth | Old freamwork genotype imputation accuracy | Standard error | New freamwork genotype imputation accuracy | Standard error |
| --- | --- | --- | --- | --- |
| 0.001X | 2.12 | 0.0074 | 73.89 | 0.0972 |
| 0.005X | 7.11 | 0.0225 | 85.83 | 0.1213 |
| 0.01X | 10.04 | 0.0270 | 88.58 | 0.1211 |
| 0.05X | 31.65 | 0.0541 | 93.09 | 0.1163 |
| 0.1X | 56.66 | 0.0849 | 95.50 | 0.1069 |
| 0.2X | 72.34 | 0.0854 | 97.36 | 0.0922 |
| 0.3X | 78.47 | 0.0784 | 98.03 | 0.0824 |
| 0.5X | 84.35 | 0.0674 | 98.57 | 0.0693 |

b

| Sequencing Depth | Old freamwork genotype imputation accuracy | Standard error | New freamwork genotype imputation accuracy | Standard error |
| --- | --- | --- | --- | --- |
| 1 | 4.89 | 0.05 | 98.29 | 0.02 |
| 5 | 11.71 | 0.09 | 98.61 | 0.03 |
| 10 | 16.78 | 0.14 | 98.66 | 0.03 |
| 30 | 36.32 | 0.14 | 98.67 | 0.03 |
| 50 | 51.35 | 0.11 | 98.48 | 0.06 |
| 100 | 67.25 | 0.17 | 98.33 | 0.03 |
| 200 | 78.35 | 0.08 | 98.03 | 0.08 |

**Supplementary Table 3. Software versions and parameter settings for SNP calling and imputation.** This table contains information on the versions and parameters of SNP calling and imputation software such as GATK, BaseVar, Stitch, and Minimac.

| Tool | Version | Parameter Settings and Command |
| --- | --- | --- |
| Fastp | 0.20.1 | fastp -i ${Sample1}_R1.fastq.gz -I ${Sample1}_R2.fastq.gz -o ${Sample1}.clean.R1.fq.gz -O ${Sample1}.clean.R2.fq.gz -h ${Sample1}.clean.html -j ${Sample1}.clean.json -w 5 |
| BWA | 0.7.5a-r405 | bwa mem -t 5 -R '@RG\tID:L01\tPL:illumina\tSM:${Sample1}' ${reference.genome}.fa ${Sample1}.clean.R1.fq ${Sample1}.clean.R2.fq \| samtools view -@ 5 -bSu - \| samtools sort -@ 5 -o ${Sample1}.sort.bam -; samtools index -@ 5 ${Sample1}.sort.bam |
| Samtools | 1.2 |  |
| GATK | 4.2.0.0 | gatk CreateSequenceDictionary -R ${reference.genome}.fa -O ${reference.genome}.dict;  gatk --java-options -Xmx300G HaplotypeCaller -I ${Sample1}.sort.bam -I ${Sample2}.sort.bam -I ${Sample3}.sort.bam -R ${reference.genome}.fa -O ${GATK.output}.vcf --native-pair-hmm-threads 50;  gatk SelectVariants -V ${GATK.output}.vcf --select-type-to-include SNP -O ${GATK.output}.snp.vcf |
| BaseVar | 0.8.0 | basevar basetype -R ${reference.genome}.fa --regions ${CHR} --batch-count 50 --max_reads 15000000 --output-vcf ${basevar_Out1_vcfgz} --output-cvg ${basevar_Out1_vcfgz} --nCPU 50 -L ${BamList} |
| Stitch | 1.6.10 | STITCH.R --chr=${CHR} --regionStart=${CHRstart} --regionEnd=${CHRend} --outputdir=${STITCH_Output} --posfile=${Posfile} --genfile=${Genfile} --bamlist=${BamList} --K=4 --nCores=50 --nGen=100 --buffer=500 --method=diploid |
| Minimac | v4.1.6 | minimac4 --compress-reference --threads 50 ${REFbcf} > ${Vcf}.msav; minimac4 ${Vcf}.msav ${input_vcfgz} -o ${minimac4_Out_vcfgz} --region ${CHR} |
